# The adaptability of Garut sheep grazing on oil palm and rubber plantations in tropical conditions of Indonesia

**DOI:** 10.14202/vetworld.2024.1889-1903

**Published:** 2024-08-24

**Authors:** Bess Tiesnamurti, Eko Handiwirawan, Santoso Santoso, Gresy Eva Tresia, Mohammad Ikhsan Shiddieqy, Achmad Fanindi, Alek Ibrahim, Endang Romjali

**Affiliations:** 1 Research Center for Animal Husbandry, Research Organization for Agriculture and Food, National Research and Innovation Agency (BRIN), Cibinong Science Center, Jl. Raya Jakarta-Bogor KM 46, Cibinong, Bogor-16911, Indonesia; 2 Animal Production Systems Group, Wageningen University & Research, De Elst 1, Wageningen 6708WD, the Netherlands

**Keywords:** botanical composition, fecal egg counts, Garut sheep, heat shock protein 70, morphometric, physiology response

## Abstract

**Background and Aim:**

The productivity of sheep in humid tropical plantation areas relies on their ability to adapt. Oil palm plantations serve as potential grazing lands for livestock. This study aimed to identify Garut sheep adaptations in oil palm and rubber plantations of the Garut district, West Java, Indonesia.

**Materials and Methods:**

The total number of sheep used was 103 as the object of this study. Each individual of sheep was used for several different observations, including: Physiological assessment using 24 sheep of various ages, molecular analysis of heat stress using 31 sheep, worm egg count using 59 sheep, and for morphological assessment using 98 sheep. A general linear model was employed to analyze rectal temperature (RT), heart rate (HR), respiratory rate, number of eggs in each gram of sample feces, body weight (BW), body condition score, and morphometric measurements according to age and sex. Forage was compared between oil palm and rubber plantations during the vegetation analysis.

**Results:**

RT, HR, and panting frequency were significantly (p < 0.05) affected by the age and sex of the sheep. The mutation was found in the coding region of the HSP70 gene, which is approximately 232 bp long. Fecal eggs were found in 99% of the fecal samples, consisting of 100% Strongyle eggs and 1.75% Moniezia eggs. BW, body condition score, shoulder height, body length, pelvic height, chest circumference, and number of eggs were significantly affected (p < 0.05) by the age and sex of the sheep. The average fresh matter of vegetation under the plantation was 248.69 ± 120.94 g/m^2^ and 718.15 ± 249.93 g/m^2^ for oil palm and rubber plantations, respectively.

**Conclusion:**

Garut sheep were adapted to oil palm and rubber plantations in the humid tropical region. Plantations are potential sources of sheep grazing and roughage. Further research is needed regarding the consumption of forages in oil palm and rubber plantations.

## Introduction

Indonesia’s archipelago is at the equator, where the adaptability of sheep in an agroecosystem significantly influences their productivity. Exposure to hot and humid climates can interfere with sheep performance, initially reducing feed intake and subsequently affecting weight gain. The relatively high ambient temperatures and strong sun exposure almost year-round in tropical regions can lead to heat stress in sheep, causing a decline in production due to reduced feed intake. Indicators include heart rate (HR), respiratory rate (RR), and rectal temperature (RT) [1–3].

Garut sheep, one of the local Indonesian breeds, has a high level of prolificacy and relatively thick wool covering its body, so it is often kept in highland areas [2, 4]. The breed exhibits an extraordinary capacity to acclimate to various environmental conditions, making them highly favorable candidates for grazing within oil palm and rubber plantations. The mutualistic association between sheep and plantation setting presents potential advantages such as sustainable land management and the enhancement of local livelihoods. With a national sheep population of 15,615,300 head and an annual slaughter rate of 1,581,373 head [5], and an increasing human population of around 1.13% per year, it is crucial to seek new sheep development areas to meet the high demand for mutton and live animals. Oil palm and rubber are the most valuable agricultural commodities in Indonesia, playing significant roles in the global market. The oil palm plantation area has expanded from 7.3 million hectares in 2008 to 16.2 million hectares in 2022, representing an annual increase of approximately 9.45%, whereas the rubber estate has grown by 1.32%, reaching 3.8 million hectares in 2023 [6].

The extensive coverage of plantations in Indonesia and the availability of forage under the estate commodities make these areas suitable for grazing. Oil palm plantations can be used as grazing areas, with various forage types, including grass, legumes, rumba, and oil palm leaf midribs, identified in different regions [7–10].

Therefore, it is significant to explore new areas for sheep production systems and determine the response of sheep raised under estate crops. Research on the adaptations of Garut sheep grazing in oil palm and rubber plantations in tropical humid regions has not been conducted extensively. The findings can support the development of local sheep and recommend using plantations as grazing areas without damaging the main plantation business, potentially increasing national sheep production. This study aims to determine whether sheep can perform well in estate crops and identify factors affecting their performance.

## Materials and Methods

### Ethical approval

The study was approved by the Research Ethics Commission, Faculty of Veterinary Medicine, Universitas Gadjah Mada (number: 002/EC-FKH/Int./2019), and the Animal Care and Use Commission, National Research and Innovation Agency (BRIN), the Republic of Indonesia (number: 204/KE.02/SK/11/2023).

### Study period and location

**Figure-1:**
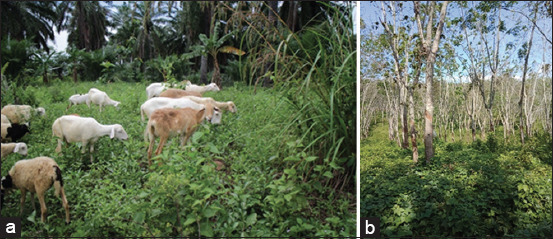
(a) Oil palm and (b) rubber plantations.

The observation was conducted during October and November 2020 for feces and blood sample collection, physiological, morphometric, parasite, and forage observations. A molecular study was conducted from November 2023 to February 2024, in order to strengthen the research. The research was carried out at the Condong Garut oil palm plantation, which has approximately 80,000 hectares, and a part of the plantation was grown with rubber (Figure-1). The site is in Pakenjeng Village, Pamulihan Sub District, Garut District, West Java Province, Indonesia. The site is located at an altitude of 180 m above sea level (7°32’13”S 107°38’56”E), with annual rainfall ranging from 35 to 592 mm and an average of 174 rainy days/year. High rainfall rates occurred in January to March and July to September had the lowest. The range of temperature was about 22°C–30°C, with the humidity from 47% to 93% [11]. Parasite observation was conducted at the Parasite Laboratory of the Indonesian Research Center for Veterinary, Indonesian Agency for Agriculture Research and Development, Ministry of Agriculture. Forage identification was conducted at the laboratory of the Research Center for Botany, Indonesian Institute of Science (LIPI). A molecular study was conducted at the Agro and Biomedical Industry Technology Development Laboratory (LAPTIAB), Agricultural Production, National Research and Innovation Agency (BRIN).

### Animals

This study used 103 Garut sheep of different ages (1–5 years) belonging to a smallholder farm that is traditionally kept. Each individual of sheep was used for several different observations, including: Physiological assessment using 24 sheep of various ages, molecular analysis of heat stress using 31 sheep, worm egg count using 59 sheep, and for morphological assessment using 98 sheep. The sheep grazed daily from 09:00 to 16:00 and did not receive other feeds afterward. Mating was conducted naturally by mixing rams with ewes in their barn; however, mating was also occur during grazing.

### Forage evaluation under a plantation

The study sites consist of oil palm and rubber plantations, where smallholder farmers graze sheep on plantation land from morning to afternoon. This study used a survey method involving direct observation of oil palm and rubber plantation areas approximately 5 and 7 years old, respectively. Plant species within the oil palm and rubber plantations were sampled using the quadrat method, as described by Hill *et al*. [12]. A total of 13 plots were established in each observation area, and understorey vegetation surveys were conducted within the rubber and oil palm plantations using randomly placed 1 m × 1 m quadrants. The distance between quadrants was 125 m. To identify plant species, the vegetation within each quadrant was cut, weighed, collected, and stored in a paper bag. The sample is then taken to the laboratory of the Research Center for Botany, Indonesian Institute of Science (LIPI), to identify the species of each forage. The absolute frequency distributions of the species were calculated using the following equation:


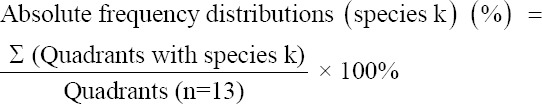


### Morphometric analysis of Garut sheep

Data were collected from 98 sheeps using an electronic scale, measuring stick, and measuring tape. The measurements were carried out in compliance with the Food and Agriculture Organization criteria [13]. Sex (male or female), age (incisors), linear body measurement (cm), including wither height (WH), rump height (RH), rump width (RW), body length (BL), chest width (CW), chest depth (CD), chest girth (CG), rump length (RL), tail length (TL), tail width (TW), right cannon circumference (CC), ear length (EL), and body weight (BW) (kg), were the quantitative parameters.

To evaluate the body proportions, breed type, and functions of the studied sheep, morphometric indices were computed. Indices were computed in compliance with Depison *et al*. [14] and Khan *et al*. [15]. The formula for calculating the index of body frame (IBF) or index of length is IBF = (BL/WH) × 100. Animals with a rectangular body frame, or longline, have this measure larger than 103; those with a square body frame, or between 97 and 103; and those with a shorter body frame, or breviglines, have this measure < 97. The formula for calculating the index of thoracic development (ITD) is ITD = (CG/WH) × 100. Animals with relative scores >120 have good thorax development. This represents the development of the thorax in animals. The dactyl thorax index (DTI), which also shows thoracic development, was calculated as DTI = (CC/CG) × 100. DTI in light animals does not exceed 10.5; in intermediate animals, it may reach 10.8; in light meat-type animals, up to 11.0; and in heavy meat-type animals, up to 11.5.

### Evaluation of physiological traits

The parameters observed were (RT, °C), (HR, breath/min), and (RR, time/min) according to the protocol presented by McManus *et al*. [16]. Before the sheep were allowed out of the pen to graze, all measurements were obtained in the morning. The number of sheep observed in this study was 24 sheeps of various ages.

#### Heat tolerance coefficient (Benezra coefficient)

The heat tolerance of 24 sheeps was calculated using the Benezra equation [17]:

HTC = (RT/39.44) + (RR/24.0)

Where:

RT: Rectal temperature (°C)

RR: Respiration rate (time/min)

39.0: Normal RT of sheep in a humid area (°C)

24.0: Normal respiration rate of sheep in a humid area (time/min)

#### Temperature-humidity index (THI)

The THI is frequently used to gauge the degree of stress caused by the climate [18, 19] using the following formula:

THI = 0.85 DBT +RH × (DBT-14.4) + 46.4

Where:

DBT: Dry bulb temperature (°C)

RH: Relative humidity (RH) in decimal form

#### Dominant coat color

The characteristics of sheep coats include dominant and striped colors. Sheep coat colors were scored by observing the dominant color distribution surrounding the body and categorized into (1) black, (2) white, (3) dark brown, (4) light brown, and (5) gray.

#### Wool cover scoring

The area of coat cover from 98 sheeps was assessed by examining the coat around the sheep. The following categories were included in the classification: 0 for totally covered in heavy wool, which includes the face and lower leg; 1 for hair only; 3 for moderate-to-heavy wool along the tops of the back, shoulders, and rump; 5 for moderate-to-heavy wool along the tops of the back, over the shoulders, and halfway down the side and rump; 7 for moderate-to-heavy wool over the body, excluding the belly and down the legs on the head; and 9 for moderate-to-heavy wool over the body, including the belly, with the exception of the top of the head, lower leg, or on the fore and rear flanks.

### Sample collection and identification of internal parasites in sheep

The parameters observed were the numbers and types of worm eggs in the sheep feces. Stool samples of 59 sheeps were collected directly from the rectum of sheep, stored in a plastic bag and tied so that it did not expose to air, and stored at 4°C before being analyzed in the Parasite Laboratory at the Indonesian Research Center for Veterinary, Indonesian Agency for Agriculture Research and Development, Ministry of Agriculture. The feces of each individual sheep were as much as 1 g and dissolved with 17 mL of water for a few minutes. After softening, the eggs of nematodes and coccidia were crushed, and 40 mL of saturated NaCl was added to float them. The filtrate was pipetted into the McMaster counting chamber until it was full and allowed to stand for 4–5 min, and the number of eggs in the McMaster boxes was counted under a microscope (Olympus CX21-Japan) at 100× magnification. Nematode eggs and coccidial oocysts were counted (in the counting chamber) under a microscope, and the numbers were multiplied by 40. The degree of infection of each worm type was expressed in eggs per gram of feces, whereas protozoan oocysts were expressed as oocysts per gram of feces.

### Segregation of the adaptation gene in sheep

#### Blood collection for DNA extraction

Blood samples (6 mL each) were collected from 131 sheep. Following the usual protocol, blood samples were drawn from the jugular vein using a venoject (Becton, Dickinson and Company, UK) containing ethylenediaminetetraacetic acid. The Genomic DNA Mini Kit (Blood/Cultured Cell) methodology (Geneaid Biotech Ltd., New Taipei City, Taiwan) was used for DNA extraction and stored at a temperature of –20^o^C until further analysis.

#### Amplification and sequencing of target DNA

The interleukin (IL)2 and heat shock protein 70 (HSP70) genes were amplified using primers (Table-1). A total volume of 25 μL containing 12.5 μL of GoTaq® Green Master Mix (Promega Corporation, USA), 1 μL of each forward and reverse primers, 1 μL of DNA template, and 9.5 μL of nuclease-free water was used for polymerase chain reaction (PCR) amplification using an ExtraGene 9600 (ExtraGene, Inc., Taiwan). The PCR reaction was run with 5 min of initial denaturation at 95°C, 10 min of denaturation at 95°C, 30 min of annealing at 60°C, 30 min of elongation at 72°C, and 5 min of final elongation at 72°C (35 cycles). The results of each fragment’s PCR were observed through 1.5% agarose gel electrophoresis, labeled with Floro Safe DNA (Axil Scientific Pte Ltd., Singapore), and recorded using a Uvitec Firereader V10 (Unit 3.05, St John’s Innovation Center, Cowley Road, Cambridge CB4 0WS-UK). HSP70 and IL2 PCR products were shipped to First Base, Singapore, for sequencing.

**Table-1:** Primer sequence for HSP70 gene and IL2 genes.

Gene	Primer sequences	Product size (bp)	GenBank accession number
HSP70	F: 5’- GGTGCTGACCAAGATGAAAG-3’ R: 5’- GTCAAAGATGAGCACGTTGC-3’	232	JN604434.1
IL2	F: 5’- GAAATCTCAGCTCTGCCATG-3 R: 5’- CCTGGACAACCAAATGGAAC-3’	336	EF056466.1

IL=Interleukin, HSP 70=Heat shock protein 70

### Statistical analysis

#### Forage evaluation under the plantation

Frequency analysis of each forage recovered from the oil palm plantation was conducted according to the percentage of species and varieties of forage found as cover crops at the plantation.

#### The morphometric measurement

The analysis of variance (ANOVA) and coefficient correlation of body size was conducted using SAS v.9.4 (SAS Institute, Inc., NC, USA). The statistical analysis uses the following model and assumption.

Y_ijk_=μ+A_i_+B_j_+ɛ_ijk_

Where:

Y_ijk_: The body size from i^th^ sex and j^th^ age of the sheep

μ: General mean

A_i_: The effect of i^th^ sex (i = 1, 2)

B_j_: The effect of j^th^ age (j = 1, 2, 3, 4)

ɛ_ijk_: Standard error from the effects of sex and age.

#### Physiological traits

Statistical analysis of physiological responses was conducted using the general linear model of SAS v.9.4 (SAS Institute, Inc.) statistical package, with the following assumptions:

Y _ijklm_=μ+A_i_+B_j_+C_k_+D_l_+ɛ_ijklm_

Where:

Y_ijklm_: The observation of physiological response from with i^th^ sex, j^th^ age, k^th^ dominant coat color, and l^th^ coat cover score

μ: General mean

A_i_: The effect of i^th^ sex (i = 1, 2)

B_j_: The effect of j^th^ age (j = 1, 2, 3)

C_k_: The effect of k^th^ dominant coat color (k = 1, 2, 3)

D_l_: The effect of l^th^ wool cover score (1 = 1, 2, 3)

ɛ_ijklm_: Standard error from the effects of sex, age, dominant coat color, and coat cover score.

#### Eggs count in feces

The statistical analysis of sheep parasites was conducted using the general linear model of SAS v.9.4 (SAS Institute, Inc.). Values of egg per gram of feces (EPG) were normalized by log (EPG+1) transformation before statistical analysis using the following model and assumption.

Y_ijk_=μ+A_i_+B_j_+ɛ_ijk_

Where:

Y_ijk_: The egg worm amounts from i^th^ sex and j^th^ age of the sheep

μ: General mean

A_i_: The effect of i^th^ sex (i = 1, 2)

B_j_: The effect of j^th^ age (j = 1, 2, 3)

ɛ_ijk_: Standard error from the effects of sex and age.

#### Amplification and sequencing of DNA

BioEdit 7.2 (https://bioedit.software.informer.com/) was used to evaluate the target sequences of HSP70 and IL2 genes. Using MEGA version 11’s Clustal W for sequence alignment, single nucleotide polymorphisms exhibiting alterations at gene sequence fragments were identified (https://www.megasoftware.net/).

## Results

### Forage evaluation under a plantation

Tables 2 and 3 present the absolute frequency distributions of understorey species in oil palm and rubber plantations, respectively. They show that plant diversity in the understoreys of rubber plantations is more varied than that of oil palm plantations. *Ischaemum muticum* and *Centrosema pubescens* Benth are two species that are generally most frequently found in cover crops under oil palm and rubber plantations.

**Table-2:** Plant diversity under oil palm plantation (n-quadrants=13).

Scientific name of the plant	Family	Absolute frequency distributions of the species (%)
*Ischaemum muticum*	Grasses/Poaceae	100.00
*Centrosema pubescens* Benth.	Leguminosae/Fabaceae	53.85
*Alysicarpus vaginalis* (L.) DC.	Leguminosae/Fabaceae	38.46
*Cyperus rotundus*	Cyperaceae	30.77
*Eragrostis tenella*	Grasses/Poaceae	23.08
*Urceola* spp.	Apocynaceae	23.08
*Phaseolus* spp.	Leguminosae/Fabaceae	23.08
*Micania micranta*	Asteraceae	15.38
*Paspalum conjugatum*	Grasses/Poaceae	15.38
*Payena acuminata* (Blume) Pierre	Sapotaceae	15.38
*Asystasia gangetica* (L.) T.Anderson	Acanthaceae	7.69
*Payena acuminata* (Blume) Pierre	Sapotaceae	7.69
*Hellenia speciosa* (J.koenig) S.R.Dutta	Costaceae	7.69
*Pueraria javanica*	Leguminosae/Fabaceae	7.69
*Axonophus compressus*	Grasses/Poaceae	7.69
*Erigeron sumatrensis* Retz.	Compositae/Asteraceae	7.69
*Melastoma malabathricum*	Melastomataceae	7.69
Miscellaneous		15.38
Biomass (gram fresh matter/m^2^)		248.69 ± 120.94

**Table-3:** Plant diversity under rubber plantation (n-quadrants=13).

Scientific name of the plant	Family	Absolute frequency distributions of the species (%)
*Ischaemum muticum*	Grasses/Poaceae	92.31
*Eragrostis tenella*	Grasses/Poaceae	84.62
*Centrosema pubescens*	Leguminosae/Fabaceae	69.23
*Triumfetta rhomboidea* Jacq.	Malvaceae	38.46
*Salvia misella* Kunth	Lamiaceae	38.46
*Phaseolus* sp.	Leguminosae/Fabaceae	38.46
*Ottochloa nodosa*	Grasses/Poaceae	30.77
*Mikania cordata* (Burm.f.) B.L.Rob.	Compositae/Asteraceae	30.77
*Imperata cylindrica*	Grasses/Poaceae	15.38
*Mimosa pudica*	Leguminosae/Fabaceae	15.38
*Stachytarpheta jamaicensis* (L.) Vahl	Verbenaceae	15.38
*Chromolaena odorata* (L.) R.M.King & H.Rob.	Compositae/Asteraceae	7.69
*Lantana camara* L.	Verbenaceae	7.69
*Tetracera scandens* (L.) Merr.	Dilleniaceae	7.69
*Urceola* spp.	Apocynaceae	7.69
*Calopogonium mucuneides*	Leguminosae/Fabaceae	7.69
*Panicum* spp.	Grasses/Poaceae	7.69
*Lepistemon binectariferus* (Wall.) Kuntze	Convolvulaceae	7.69
*Wallastonia biflora* (L.) DC.	Compositae/Asteraceae	7.69
*Cyathula prostrata* (L.) Blume	Amaranthaceae	7.69
*Andrographis paniculata* (Burm.f.) Nees	Acanthaceae	7.69
*Payena acuminata* (Blume) Pierre	Sapotaceae	7.69
*Urena lobata L.*	Malvaceae	7.69
Miscellaneous		23.08
Biomass (gram fresh matter/m^2^)		718.15 ± 249.93

We found 17 different types of natural vegetation that was used as cover crops in the oil palm field under investigation. Out of the 17 plants identified, we identified four grasses (Poaceae) species and four leguminosae (Fabaceae) species. Meanwhile, 23 species comprising natural vegetation were identified in the understorey of the rubber plantation. Of the 23 identified species, we identified five grasses (Poaceae) species and four leguminosae (Fabaceae) species. The absolute frequency distribution of species in the understorey of oil palm plantations of more than 50% was in the species *I. muticum* and *C. pubescens Benth*, occurring in 100% and 53.85% of the sampled quadrants, respectively. Moreover, in the understorey of rubber plantations, species with an absolute frequency distribution of more than 50%, including *I. muticum, Eragrostis tenella*, and *C. pubescens Benth*, occurred in 92.31%, 84.62%, and 69.23% of the sampled quadrants, respectively.

**Figure-2:**
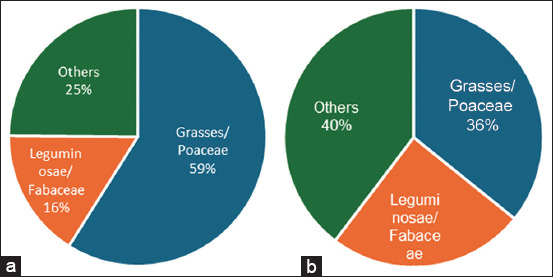
Fresh matter forage under (a) oil palm and (b) rubber plantations.

The largest proportion of total biomass collected in the understorey of palm oil plantations was grasses (59%), followed by leguminous (16%), and other plants (25%), as illustrated in Figure-2. Meanwhile, grasses contributed 36%, leguminous plants contributed 25%, and other plants contributed 40% of the biomass in the understoreys of the rubber plantations.

### Sheep morphometric performance

Table-4 presents the average BW and 12 body sizes of sheep kept in the plantation areas by sex and age. In general, male sheep have a larger BW and body size than female sheep, but this difference was not significant. The results of the ANOVA show that age had a significant effect on BW and several body sizes, namely WH, RH, BL, CD, and CG, whereas the other body sizes did not differ significantly. The BWs and measurements of RH, CD, and CG of 1-year-old sheep were significantly different from those of 2-, 3-, and 4-year-old sheep, whereas these measurements for 2-, 3-, and 4-year-old sheep were not significantly different. Meanwhile, the WH and BL measurements at the age of 1 year are significantly different from those at the age of 3 and 4 years.

**Table-4:** Least square means of body weight and size of sheep kept by farmers in plantation areas.

Body size (cm)	Sex (n)				Age (n)							
	
	Female (71)		Male (11)		1 ≤ 2 years (13)		2 ≤ 3 years (15)		3 ≤ 4 years (29)		≥ 4 years (25)	
					
	Mean	SE	Mean	SE	Mean	SE	Mean	SE	Mean	SE	Mean	SE
WH	58.8	0.8	59.7	1.9	55.5^a^	1.8	59.6^ab^	1.8	61.5^b^	1.4	60.3^b^	1.5
RH	62.1	0.8	61.5	1.8	57.0^a^	1.7	62.9^b^	1.7	63.2^b^	1.3	63.9^b^	1.4
HW	11.3	0.3	11.3	0.8	10.8	0.7	10.8	0.7	12.0	0.6	11.7	0.6
BL	52.4	0.7	52.1	1.8	49.3^a^	1.6	52.6^ab^	1.7	53.7^b^	1.3	53.4^b^	1.4
CW	12.0	0.4	11.5	1.0	11.5	0.9	11.2	0.9	11.9	0.7	12.4	0.8
CD	21.7	0.5	22.6	1.1	19.6^a^	1.1	22.7^b^	1.1	23.6^b^	0.8	22.9^b^	0.9
CG	67.5	0.9	68.6	2.2	62.8^a^	2.0	67.8^b^	2.0	71.0^b^	1.6	70.6^b^	1.7
RL	18.0	0.4	18.0	0.9	18.0	0.9	17.1	0.9	18.2	0.7	18.7	0.8
TL	16.9	0.6	16.3	1.4	17.1	1.3	15.4	1.3	16.8	1.0	16.9	1.1
TW	5.4	0.2	5.5	0.4	4.9	0.4	5.6	0.4	5.8	0.3	5.4	0.3
CC	8.2	0.2	7.2	0.6	7.7	0.5	7.4	0.5	7.8	0.4	7.6	0.5
EL	9.6	0.4	11.5	0.9	9.0	0.9	11.0	0.9	10.6	0.7	11.3	0.8
BW	22.7	0.6	25.5	1.5	16.8^a^	1.4	25.4^b^	1.4	27.3^b^	1.1	26.8^b^	1.2

SE=standard error; ^a,b^ different superscripts on the same row for sex or age indicate significant differences (p < 0.05), WH=wither height, RH=rump height, HW=hip width, BL=body length, CW=chest width, CD=chest depth, CG=chest girth, RL=rump length, TL=tail length, TW=tail width, CC=right cannon circumference, EL=ear length, BW=body weight

Table-5 presents the overall phenotypic correlations among all morphometric metrics. With the exception of the very weak and non-significant connection between EL and all body sizes, as well as between BW and TL, TW, and CC, most of these correlations were positive and statistically significant (p < 0.001). The two pairs with the highest correlation were BL and CD (0.75) and WH and RH (0.85). The correlations between CC and WH (0.30), TL and WH, RH (0.28 and 0.29), BW and RW, CW, RL, TL, TW, CC, and EL (0.28, 0.27, 0.29, 0.07, 0.20, 0.15, and 0.12, respectively), and EL and all body sizes (correlation range 0.01–0.20) were all rather low (≤0.3).

**Table-5:** Pearson’s correlation coefficients (above diagonal) and significance (below diagonal) among the body measurements of sheep kept by farmers.

Body size	WH	RH	RW	BL	CW	CD	CG	RL	TL	TW	CC	EL	BW
WH	1.00000	0.84557	0.56172	0.54018	0.56853	0.71388	0.57324	0.54530	0.28453	0.41274	0.30021	0.03450	0.71844
RH	<0.0001	1.00000	0.60148	0.64110	0.54050	0.64106	0.64843	0.58077	0.29586	0.36519	0.35618	0.16627	0.67594
RW	<0.0001	<0.0001	1.00000	0.60393	0.65223	0.57090	0.61851	0.52163	0.47238	0.50670	0.54065	0.16981	0.28961
BL	<0.0001	<0.0001	<0.0001	1.00000	0.46660	0.75055	0.71740	0.43036	0.38939	0.37240	0.49591	0.20298	0.39191
CW	<0.0001	<0.0001	<0.0001	<0.0001	1.00000	0.63588	0.65393	0.68469	0.57505	0.61237	0.45802	0.02262	0.27427
CD	<0.0001	<0.0001	<0.0001	<0.0001	<0.0001	1.00000	0.73104	0.48693	0.41328	0.48913	0.40834	0.13260	0.54780
CG	<0.0001	<0.0001	<0.0001	<0.0001	<0.0001	<0.0001	1.00000	0.67950	0.51552	0.48339	0.52297	0.16997	0.48519
RL	<0.0001	<0.0001	<0.0001	<0.0001	<0.0001	<0.0001	<0.0001	1.00000	0.51798	0.55425	0.46261	0.04361	0.29099
TL	0.0096	0.0070	<0.0001	0.0003	<0.0001	0.0001	<0.0001	<0.0001	1.00000	0.47558	0.47574	0.04092	0.07637
TW	0.0001	0.0007	<0.0001	0.0006	<0.0001	<0.0001	<0.0001	<0.0001	<0.0001	1.00000	0.34051	0.01128	0.20860
CC	0.0061	0.0010	<0.0001	<0.0001	<0.0001	0.0001	<0.0001	<0.0001	<0.0001	0.0017	1.00000	0.14409	0.15664
EL	0.7583	0.1355	0.1272	0.0674	0.8401	0.2350	0.1269	0.6972	0.7151	0.9199	0.1965	1.00000	0.12828
BW	<0.0001	<0.0001	0.0083	0.0003	0.0126	<0.0001	<0.0001	0.0080	0.4953	0.0600	0.1599	0.2507	1.00000

WH=Wither height, RH=Rump height, BL=Body length, CW=Chest width, CD=Chest depth, CG=Chest girth, RL=Rump length, TL=Tail length, TW=Tail width, CC=Right cannon circumference, EL=Ear length, BW=Body weight

According to the IBF values determined, sheep raised by farmers in plantation areas had breviglines or short frames. Measured ITD values were crucial factors in estimating body conformation. Sheep kept by farmers in the plantation area had a value of 116.28 ± 13.06, which means that they did not have good thorax development. Meanwhile, the calculation results obtained a DTI value of 11.55 ± 2.31, which means that the sheep were categorized as heavy meat-type animals (Table-6) [14, 15].

**Table-6:** Body index of sheep kept by farmers in oil palm plantation areas.

Index	Value	Category
Index of body frame (IBF)	89.18 ± 10.41	Animals with a rectangular body frame, or longline, have this measure larger than 103; those with a square body frame, or between 97 and 103; and those with a shorter body frame, or breviglines, have this measure< 97 [14, 15]
Index of thorax development (ITD)	116.28 ± 13.06	Animals with relative values greater than 120 exhibit well-developed thoraxes [14, 15].
Dactyl thorax index (DTI)	11.55 ± 2.31	DTI in light animals does not exceed 10.5; in intermediate animals, it may reach 10.8, in light meat-type animals, up to 11.0, and in heavy meat-type animals, up to 11.5 [14, 15]

### Evaluation of sheep physiological traits

**Figure-3:**
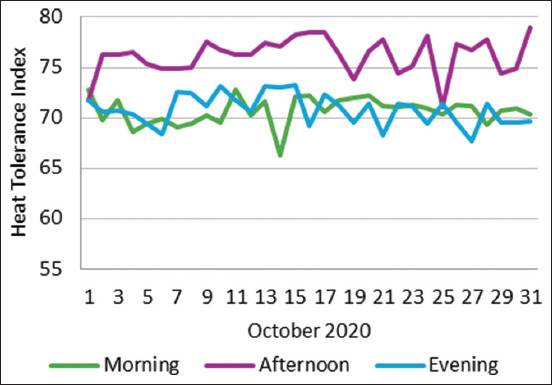
Temperature-humidity index (THI) in the morning, afternoon, and evening of the study site (October 2020). Source: Recalculated Data from the National Climatology Station of Pamengpeuk, West Java.

The average THI during October 2020 was 70.67, 76.15, and 70.81 for morning, afternoon, and evening observations, respectively (Figure-3). The range of THI in the morning, afternoon, and evening from this study was 66.28 to 72.79, 71.07 to 78.51, and 67.72 to 73.18, respectively.

**Figure-4:**
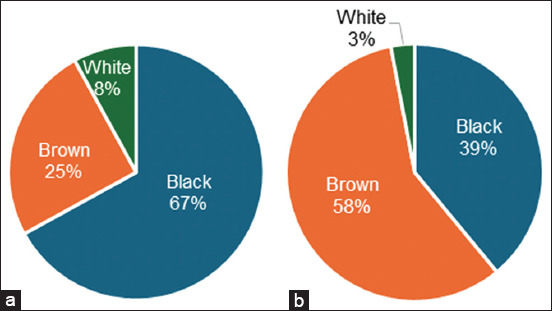
The proportion of dominant coat colors in (a) male and (b) female sheep.

Among the various coat colors segregated in sheep, males had a higher percentage of black (67%), followed by white (25%) and brown (8%). However, the coat color distribution in females is controversial, where the dominant coat color was white (58%), followed by black (39%) and brown (3%) (Figure-4).

**Figure-5:**
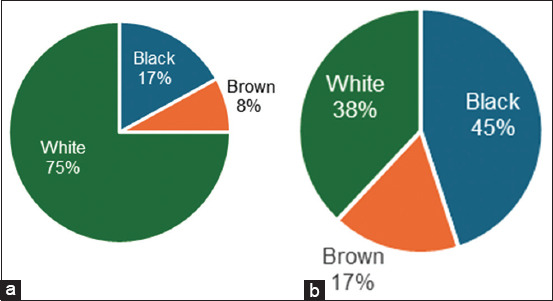
Proportion of striped color in (a) male and (b) female sheep.

Not all sheep breeds had segregation of stripes or patches on their coat covers; however, the Garut sheep in this study consisted of three stripe colors with different percentages for males and females (Figure-5). The stripe distribution of males consisted of white (75%), black (17%), and brown (8%), whereas females had a different percentage of stripe color, black (45%), followed by white (38%) and brown (17%).

Table-7 presents the influence of sex, age, dominant coat cover, and wool cover score on the physiological response. RT was significantly influenced (p < 0.05) by sex, whereas HR, RR, and HTC were not influenced (p > 0.05) by age and sex, respectively. The mean RT in this study was 38.59°C with a range of 37.5°C–39.8°C whereas the mean RR, HR, and HTC were 44.24 breath/min, 83.15 time/min, and 1.94, respectively. Likewise, the dominant coat color and wool cover score of sheep did not have any influence on HR, RR, and HTC, respectively.

**Table-7:** Least square mean physiological responses.

Source of variation	n	Heart rate (Time/min)		Respiration rate (Breath/min)		Rectal temperature (°C)		Heat tolerance coefficient	
			
		Mean	SE	Mean	SE	Mean	SE	Mean	SE
Sex									
Male	7	85.90^a^	3.22	43.09^a^	3.14	37.93^a^	0.36	1.88^a^	0.11
Female	78	82.47^a^	0.95	44.53^a^	0.93	38.68^a^	0.10	1.95^b^	0.03
Age (year)									
≤ 1	41	85.53^a^	1.72	43.44^a^	1.68	38.21^a^	0.19	1.90^a^	0.07
2	25	84.78^a^	2.32	43.59^a^	2.26	38.27^a^	0.26	1.91^a^	0.09
≥3	19	82.25^a^	2.38	44.40^a^	2.32	38.43^a^	0.26	1.94^a^	0.09
Dominant color of the coat									
White	45	77.70^a^	2.89	43.36^a^	2.88	38.24^a^	0.32	1.90^a^	0.12
Brown	3	81.33^a^	5.46	43.05^a^	5.44	37.53^a^	0.62	1.89^a^	0.22
Black	37	80.94^a^	2.83	44.69^a^	2.82	38.46^a^	0.32	1.96^a^	0.11
Wool cover score									
1	6	83.21^a^	3.63	43.06^a^	3.62	38.36^a^	0.41	1.89^a^	0.15
3	36	84.46^a^	2.41	41.91^a^	2.4	38.15^a^	0.27	1.84^a^	0.10
5	43	84.23^a^	2.62	44.46^a^	2.61	37.97^a^	0.29	1.95^a^	0.10

SE=Standard error, ^a,b^different superscript letters on the same column for sex, age, dominant coat color, and wool cover score indicate significant differences (p< 0.05)

### Sheep infection due to helminthiasis and coccidiosis

The laboratory analysis results showed that all the observed sheep (100%) were positively infected with gastrointestinal nematodes and coccidiosis. The mean number of eggs per gram of feces/EPG for all sheep infected with gastrointestinal parasites was 1.331. The results of the analysis indicated that the amount of EPG in the feces of male and female sheep did not differ significantly (Table-8).

**Table-8:** Least square mean eggs per g of feces (EPG) of gastrointestinal parasites in sheep.

Variable	n	Mean	SE	Geometric means
Sex				
Female	49	2.60^a^	0.10	1.429
Male	7	2.35^a^	0.22	1.447
Age (year)				
1	15	2.40^a^	0.17	1.102
2	28	2.56^a^	0.16	1.324
3	13	2.46^a^	0.19	1.614

SE=Standard error, the same superscript in the same column for gender and age shows no significant difference (p > 0.05)

A total of 56 sheep from nine farmers found that all sheep were positively infected with gastrointestinal parasites (60.8% *Strongyla*. 38.9% *Coccidiosis*, and 0.30% *Moniezia*). Strongyloides and *cestodes* worm eggs were not found in the observed sheep fecal samples (Table-9). The mean number of coccidia oocysts was 841.7.

**Table-9:** Mean number of eggs per gram of feces (EPG) according to the types of gastrointestinal parasites in sheep.

Types of parasitic eggs	Number of samples	EPG	Prevalence (%)
*Strongyle*	56	489.0	60.8
*Coccidiosis*		841.7	38.9
*Moniezia*		1.6	0.3

### Amplification of primers related to adaptation

**Figure-6:**
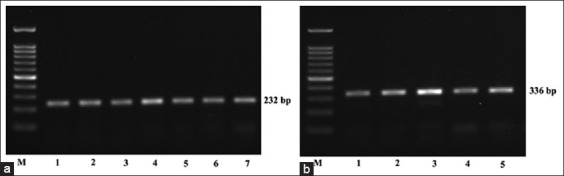
Electrophoresis results from polymerase chain reaction (PCR) products of heat shock protein 70 (HSP70) and interleukin (IL)2. (a) PCR products of HSP70 and (b) IL2. M = 100-bp marker; lines 1–5/7 represent Garut sheep samples.

**Figure-7:**
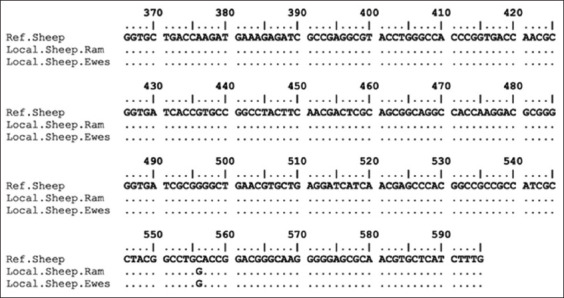
Nucleotide sequence alignment of the heat shock protein 70 gene-coding region in Garut sheep samples, including standard, ram, and ewe samples.

**Figure-8:**
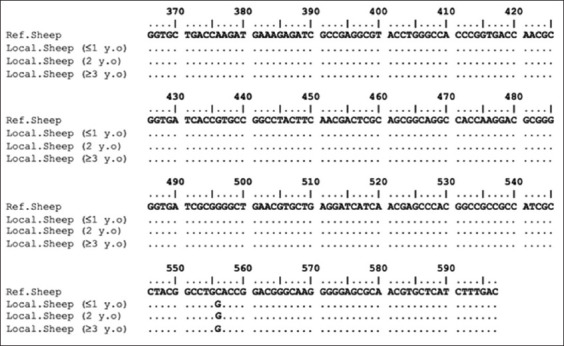
Nucleotide sequence alignment of the heat shock protein 70 gene-coding region in Garut sheep samples, including standard, 1-year, 2-year, and 3-year-old samples.

The PCR products of HSP70 and IL2 were successfully amplified (Figure-6). Mutation (g.556C > G) and deletion (g516-520) were detected in the coding region of HSP70, which was 232 bp in length, as shown in Figures-7 and 8. This study successfully amplified the 336 bp region of the IL2 gene at positions 1244–1579 (EF056466.1). However, no polymorphism was found at the IL2 target position among all aligned samples.

## Discussion

### Forage composition in plantations

Results showed that the botanical composition of cover crops under oil palm and rubber plantations in our research site consisted of grass, legumes, and other vegetation, including weeds. The grass family is most commonly found in the understorey areas of oil palm and rubber plantations, accounting for 23.53% and 21.74%, respectively. In addition, legumes were also prevalent in the understorey areas of oil palm and rubber plantations, constituting 23.53% and 17.39% of the total, respectively. Most of the vegetation in the plots consisted of species such as *I. muticum, E. tenella*, and *Centrosema pubescent*. Several studies have reported finding 17–45 plant species understoreys of oil palm plantations [20–24]. Meanwhile, vegetation observed in rubber plantations contained 20–25 plant species [20, 25]. Furthermore, Kanny *et al*. [20] identified 29 species in oil palm plantations located in Musi Banyuasin Regency, South Sumatra Province, Indonesia, which were dominated by *Ottochloa nodosa* (11.92%) and *Asystasia gangetica* (11.40%). The rubber plantations were dominated by *Cynodon dactylon* (28.42%) and *Panicum repens* (9.20%), respectively. The grasses *A. compressus, O. nodosa, C. oxyphyllum, A. hispidus*, and the fern *Adiantum latifolium* (*Pteridaceae*) were the most frequently occurring species in a study by Grinnell *et al*. [21], appeared in 72%, 71%, 51%, 28%, and 21% of the studied quadrats, respectively. The authors conducted their study at an oil palm plantation in the state of Negeri Sembilan, Peninsular Malaysia, in the district of Pedal. Variations in plant adaptation and ecosystem characteristics may account for the disparity in the diversity of vegetation species. Sahari *et al*. [24] demonstrated that the composition of understorey species in oil palm plantations varied across different age ranges, including 2–6, 9–11, and 17–21 years old, respectively. Similarly, variations in the understorey species were also observed in the rubber plantations. There were 23 species in rubber agroforestry and 15 species in rubber monoculture, according to Muhdi *et al*. [25]. With significant value indices of 30.64% and 33.01%, *Stachytarpheta jamaicensis* and *A. gangetica* were dominant understorey species in rubber agroforestry and rubber monoculture, respectively.

Furthermore, when all 13 quadrants are combined, the biomass understorey of the rubber plantation is higher (3,328 g of fresh matter) than that of the oil palm plantation (1,847 g of fresh matter). Nonetheless, compared to rubber plantations, the percentage of biomass grasses in oil palm plantations was higher. This indicates that the potential for forage for sheep was 75% and 61% in oil palm and rubber plantations, respectively. If calculated per hectare, the biomass potential is estimated to be approximately 1,065 and 1,561 kg of fresh matter, respectively. With an average weight of 40 kg per sheep, approximately 4 kg of fresh forage per day would be required (10% of BW). Grinnell *et al*. [21] reported that the total collected biomass was 743.2 g dry matter (DM) from 72 quadrats (0.5 m × 0.5 m), the highest was for *O. nodosa* (362.4 g DM), followed by *A. compressus* (149.6 g DM), and *C. oxyphyllum* (80.7 g DM). In the meantime, the average biomass of understorey plants in rubber agroforestry was 0.84 tons/ha, whereas that in rubber monoculture was 0.94 tons/ha [25]. The site of our study has a high level of vegetation that is not categorized as legumes or forage, some of which may be weeds. Heavy weed vegetation can cause overgrazing of pastures and a lack of proper knowledge about pasture management [26]. Farmers use cover crops as fodder and plantations for sheep grazing on grassland. However, farmers may not maintain rotating pastures; instead, they may simply use vegetation understories without supplementation. Other factors that contribute significantly to weed growth include insect application, nutrient levels, and climate [26].

### Sheep morphometric performance

The increase in BW and the development of body size, RH, CD, and CG have almost reached a peak at the age of 2 years. Afterward, BW continued to increase; however, the development of body size in RH, CD, and CG slowed down. Based on this, at the age of 2 years, it is assumed that the sheep will reach maturity. In comparison to females, males had a higher mature weight, whereas females matured at a faster rate and reached adulthood earlier [27]. This is due to the influence of hormonal secretion, as reported by Wassie *et al*. [28] revealed that prenatal androgen therapy enhances the birth weight of lambs, which is attributed to the impact of hormonal production. However, the ANOVA results showed that the sex variable had no effect on the BW and size of the sheep, as also reported by Washaya *et al*. [29] that the BW (birth weight and weaning weight) of males and females of several sheep breeds were not significantly different, as found in this study. Sheep growth patterns can be modeled using BW data, allowing for the prediction of BW at any age [27, 30].

Animal genetic resources’ phenotypic characterization includes quantitative characteristics for body measurements. The process of identifying various breed populations and defining their external and production features in a given habitat under certain management while accounting for the social and economic factors that impact them is known as phenotypic characterization of animal genetic resources [13].

Animal body measurements are often used to compare differences in size and form [19] and to estimate body and carcass weights [31, 32]. The IBF findings categorized Garut sheep as brevigiline [18, 19]. An animal is considered longline if its body frame is rectangular; it is considered breviglines if its body frame is square; and it is considered short if its body frame is less than 97. However, animals with relative ITD levels exceeding 120 exhibit healthy thoracic development [18, 19]. DTI was discovered to be no more than 10.5 in light animals; in intermediate animals, it may be as high as 10.8, in light meat-type animals, up to 11.0, and in heavy meat-type animals, up to 11.5 [18].

Body structural indices can explain differences in breed type and function among goats [33]. Animal body components were described by their proportions using morphometric indices. Body measurements can be used to differentiate populations and estimate genetic distances among groups or breeds of livestock. Several researchers have reported the use of body size to estimate the genetic distance between sheep breeds [34, 35]. Several techniques have been developed to predict BW in sheep, including computer vision techniques [36], artificial intelligence techniques [37], and artificial neural network algorithms [38]. Suppose there is a strong correlation between the phenotypic characteristics of one measurement and another. In that case, one body size can be used as an estimate of the other, as reported previously by Djaout *et al*. [31] and Bautista-Díaz *et al*. [32]. For example, the WH size can be used to estimate the RH size and vice versa, and the same applies to the BL and CD sizes. Therefore, BW can be estimated based on WH measurements.

Compared with animals with lighter coats, those with darker coats absorb more heat radiation and are more vulnerable to heat stress [3]. According to Borthwick *et al*. [39], individual animals react to environmental conditions brought about by climate change, which impacts their social connections and the group’s social network. Sheep housed among rubber and oil palm trees, however, might not be subjected to heat stress because they are shaded by palm trees.

### Physiological response of sheep

Sheep have been grazing since they were born as part of their management practices and because of the availability of forage surrounding the farmers. RT and RR are the main mechanisms of thermoregulation associated with heat stress and can be used as relevant measures of body temperature; therefore, they can be used as parameters indicating heat stress in sheep. These results are in agreement with a previous report by Gonzaga dos Santos *et al*. [40] for an RT range of 38.5°C–39.7°C. The mean HR, RR, and RT from this study are in agreement with the previous report by Salih *et al*. [41] for the Sudan desert, where the mean RR and RT were 41.3 time/min and 38.6°C, respectively. Similarly, our findings are also consistent with the report of Aboul Naga *et al*. [42] on Wahiti sheep in Egypt as well as Ruiz-Ortega *et al*. [43] on Blackbelly adult Ewe and lambs in Mexico. Likewise, ewes from integrated crop-livestock systems developed in semi-arid Brazil [44] had lower HR and RR than ewes kept in barns. Similarly, Mayasari *et al*. [45] reported that the RR and HR of Garut sheep kept outdoors were lower than those of in-door sheep. These results indicate that Garut sheep can adapt to oil palm and rubber plantations, considering that the RT is still below 40°C.

The non-significant influence of age on HR, RR, RT, and HTC could be due to the well-adaptability of sheep to the surrounding comfort zones of the plantation areas reflected in the sheep’s physiological responses. On the other hand, Abbaya *et al*. [46] reported that age significantly influenced RT (38.31°C vs. 39.03°C) and HR (39.3 vs. 33.0 breath/min) in young and adult sheep, respectively. In addition, age did not contribute significantly to the effects of RR (58.83 vs. 64.67 time/min) and HTC (3.56 vs. 3.83) in young and adult sheep, respectively. This result is in line with the findings of our study. HTC can be expressed as the adaptability of an individual to the environment, where RT and respiration rate are the main contributors to the coefficient. Our study conformed to Mayasari *et al*. [45], who reported that an intensive barn with outside access improves sheep’s physiological adaptability as well as increases the performance of Garut ewes.

The temperature and RH values in the environment were used to compute THI, which is typically used to assess heat stress. It can be divided into three categories: mild heat stress (THI 72–79), moderate heat stress (THI 79–89), and severe heat stress (THI >89) [47]. Our THI calculation result was similar to that of Ruiz-Ortega *et al*. [43], who studied Blackbelly ewes and lambs in Southern Mexico throughout the summer under tropical conditions. The THI values were 79 U in the morning, 88 U in the afternoon, and 77 U at night. Their THI scores varied from 77 to 90 units (U). In our study, sheep were considered to be under moderate heat stress in the afternoon (by looking at THI, which is between 72.0 and 78.0 U); however, in the evening and the next morning, animals adapt to the microclimate changes resulting in the absence of heat stress. The influence of the afternoon THI did not harm the grazing sheep because they were herded from 09:00 to 16:00 under the canopy of the plantation trees, which protected their exposure to direct sunlight. In general, the THI in that region was considered favorable and supported sheep raising.

The wool cover score also did not significantly contribute to HR, RR, RT, and HTC. This is because the sheep’s wool cover in this observation mostly consisted of hair only over the body; sheep had moderate to heavy wool along the tops of their backs, shoulders, and rump, and some of them had moderate to heavy wool along the tops of their backs, over shoulders, and halfway down and rump. This is important, considering that the type of wool used in sheep greatly influences skin and RT. Garut sheep are a mixture of local sheep, merino sheep, and fat-tailed sheep that began crossbreeding around 1800. Therefore, the wool quality of this sheep is considered coarse, with a mixture of hair and wool. In contrast, a previous study by Panjono *et al*. [48] using Javanese thin and fat tails showed that wool shearing significantly influences the physiological response, behavior, and production traits. Wool shearing in sheep significantly reduced RR, HR, and RT and increased feed intake compared with non-shearing sheep.

### Sheep infection due to helminthiasis and coccidiosis

The results of the examination of sheep feces found a form of cyst known as an oocyst whose distribution is supported by environmental conditions [49–51]. Plantation areas are generally ideal conditions for protozoa development, which could be due to their humidity. Diseases caused by protozoa are more serious in lambs aged 4–6 months or cattle with intensive livestock systems. As a result, environmental factors and maintenance practices are crucial in the development of coccidiosis in small ruminants [52].

Likewise, the age of the sheep did not differ significantly (Table-8), which is in line with the results reported by Mahlehla *et al*. [53]. The level of immunity to gastrointestinal nematode infection is affected by age, nutritional status, and genotype [54–57]. The sheep observed in this study were adult sheep aged >1 year, so it is suspected that an immune process has developed. Overall, the degree of Strongyles infestation was included in the low category, and the degree of coccidiosis infestation was moderate [53, 58].

The low average number of eggs/oocysts indicates that the degree of infestation in sheep is low, with mild clinical symptoms. However, most coccidia (*Eimeria* spp.) infested in small numbers will produce subclinical symptoms [58–61]. In general, sheep are naturally infected with low levels of infestation following long exposure, which will result in disturbances in growth and immune processes [62].

Most sheep are reared by farmers using a semi-intensive rearing system. The sheep grazed in the oil palm plantation area around 09.00–16.00, and it is suspected that internal parasite larvae decreased when the sheep grazed. Nematode worm eggs do not require an intermediate host; thus, if they are in an ideal location, they can hatch quickly and develop into infective larvae within 24 h [63, 64]. The infective stage of third-stage larvae (L3) of some nematodes can move vertically toward the tops of grasses or plants. These events occur in the morning, and their frequency decreases in the presence of ultraviolet light [65]. Therefore, grazing management is crucial to reduce internal parasite infestations in sheep, which is in line with tropical climate conditions with high temperatures and humidity throughout the year and encourages the development of gastrointestinal nematodes [66]. Many *Strongyle* spp. worm larvae die from exposure to hot weather in the dry season and reproduce rapidly in the wet season [67]. Worm larvae can penetrate the skin of endoparasites and enter the veins, then move to organs other than digestion and enter respiratory organs [68]. Strongyle worm infection spp. can affect the BW of livestock [69]. Unlike *Monieza* spp. worms, which require oribatid mites to complete their life cycle, transmission occurs through the ingestion of cysticercoid-infected oribatid miles [70]. Investing in young and adult worms can cause intestinal irritation, leading to digestive disorders. Severe infections are related to the number of oribatid mites in grasslands [71]. The number of mites will be high in pastures with continuous grazing intensity for a long time. Mites have a habit of rising at night or dusk in the dark parts of the grass and, during the day, hiding on the grass floor that is not reached by light or on the ground (negative phototropism) [72].

### Segregation of adaptation genes in Garut sheep

The present findings did not reveal any polymorphism of HSP70 and IL2 markers, which is inconsistent with a previous study by Rawash *et al*. [73]. Their research showed that HSP70 could be used as a breeding reference to determine the genetic potential and adaptability of Egyptian Barki sheep. Furthermore, Younis [74] confirmed that the HSP70 gene was expressed in Abu Dlik and Barki sheep in Egypt, and a higher mRNA level of HSP70 was detected in Abu Dlik. Another approach to investigate the expression of stress genes in sheep was conducted by measuring HSP70 levels. In Pakistan, Barki sheep had a much higher HSP70 than Damani sheep. These two groups also showed a strong correlation with physiological indicators, including HR, RR, and RT [75].

There may be no polymorphism of the molecular markers HSP70 and IL2 because the sheep in this observation came from the same parental group. It is possible that uncontrolled mating occurs during grazing when ram and ewe are grazing in the same area. Another possibility is that the HSP70 and IL2 fragments tested were not specific; therefore, no polymorphism was observed.

If stress is identified in a subpopulation, there are ways to overcome it, such as identifying existing breeds, especially using local or indigenous breeds that are adapted to climatic and nutritional stress, and identifying genes that are responsible for stress [76]. Not to be forgotten, the animal management system has to be improved, such as providing adequate shade surrounding the animal facilities, improving the management of grazing lands, reducing livestock numbers, and improving the management of grazing lands.

## Recommendations

An abundant area of estate crops, mainly oil palm and rubber, can be used as pasture for sheep, so grazing management has to accommodate the management of the main crops. Forage varieties can be introduced to the plantation to enhance feed quality by considering the shade from these trees. In oil palm plantations, when the canopy has not yet been covered and the plants are still young (up to 3 years), grazing is not recommended to avoid damaging the main trees. However, when the trees have grown taller (4–20 years old), and the canopy is not fully covered, the area can be used for sheep grazing.

The management of sheep mating, weaning, and rearing must take into account the availability of forage and the application of grazing rotation. Ram rotation for mating to prevent inbreeding must also be considered. Business units that can be developed in oil palm-livestock integration or rubber livestock integration systems include producing feeder lambs and fattening.

The location of the plantation, the age of the trees, and the kind and availability of forage all affected the oil palm plantation’s carrying capacity. According to this study, the estimated fresh matter generated by rubber and oil palm plantations was 1561 and 1065 kg per ha, respectively. With an average weight of 40 kg per sheep, approximately 4 kg of fresh forage per day would be required (10% of BW). The estimated production and non-production oil palm plantations are approximately 11,750,000 and 4,100,000 ha, respectively [6]. Therefore, the carrying capacity of oil palm for sheep production in Indonesia is predicted to be 3,128,437,500 head per year.

## Conclusion

The forage composition under the oil palm and rubber plantations was more varied. This Garut sheep’s index body frame and DTI were considered short and heavy meat-type animals. The adaptation of sheep to grazing systems on oil palm and rubber plantations has not shown any heat stress, as seen from HR, respiration, and RT, which are within the normal range for sheep. Internal parasitic infection in grazing sheep showed that there were *Coccidiasi*s and *Strongyla* in sheep feces, with the category of mild infestation. There was no evidence of polymorphism of the primers HSP70 and IL2 in the sheep under this study.

## Authors’ Contributions

BT and ER: Designed and supervised the study and prepared and revised the manuscript. EH and MIS: Collected and analyzed the data, performed fieldwork, and prepared and revised the manuscript for physiological and morphometric traits aspects. GET and AF: Collected and analyzed the data, performed fieldwork, and revised the manuscript for the forage in plantation aspect. AI and SS: collected and analyzed the data, performed laboratory work, and prepared and revised the manuscript for molecular and health aspects. All authors have read, reviewed, and approved the final manuscript.
